# Cost-utility analysis of lurasidone for the first-line treatment of schizophrenia in China

**DOI:** 10.3389/fpubh.2022.987408

**Published:** 2022-09-15

**Authors:** Jia Liu, Lidan Cao, Jing Wu

**Affiliations:** ^1^School of Pharmaceutical Science and Technology, Tianjin University, Tianjin, China; ^2^Center for Social Science Survey and Data, Tianjin University, Tianjin, China

**Keywords:** cost-utility, lurasidone, olanzapine, risperidone, schizophrenia

## Abstract

**Objective:**

To evaluate the cost-effectiveness of lurasidone compared with olanzapine and risperidone in the first-line treatment of patients with schizophrenia from a Chinese healthcare system perspective.

**Methods:**

A Markov model with 6-week cycle was constructed to reflect the disease progression of schizophrenia patients in the acute and maintenance phase. Probabilities of treatment discontinuation and adverse events in the acute phase were derived from the 6-week lurasidone clinical trial and a published network meta-analysis; long-term risks of relapse and discontinuation were estimated based on the 12-month lurasidone clinical trial and other treatment comparison studies. Cost inputs were derived from published literature and Chinese official documents, supplemented by expert opinions when necessary. Utility values were taken from published literature. Costs and quality-adjusted life-years (QALYs) were assessed over 15 years with a discount rate of 5% per year.

**Results:**

Over a 15-year time horizon, lurasidone yielded an improvement of 0.197 QALYs with a cost saving of CN¥12,093 (US$1,753) vs. olanzapine and an improvement of 0.116 QALYs with a cost saving of CN¥6,781 (US$983) vs. risperidone. One-way sensitivity analyses demonstrated robust base-case results since all analyses yielded net monetary benefits >0 at a willingness-to-pay threshold of CN¥72,447.00 (US$10,499.57)/QALY. Probabilistic sensitivity analyses suggested that lurasidone had 99.7, 99.9, and 100% probability of being cost-effective vs. olanzapine and risperidone at the conventional decision thresholds of 1, 2, and 3 times the Chinese per capita gross domestic product [namely CN¥72,447.00 (US$10,499.57)/QALY, CN¥1,44,894.00 (US$20,999.13)/QALY, and CN¥2,17,341.00 (US$31,498.70)/QALY in 2020], respectively.

**Conclusion:**

Treatment with lurasidone was predicted to improve health outcomes and be a dominant strategy for patients with schizophrenia, compared with olanzapine and risperidone, in China.

## Introduction

Schizophrenia is a chronic and severely debilitating mental disorder with unknown etiology, which is characterized by high morbidity, high recurrence rate, high disability rate and heavy socio-economic disease burden (1). This disease affected approximately 23.6 million people worldwide and generated a humanity burden with a total of 15.1 million years lived with disability (YLDs) in 2019 (2). A systematic review informed that annual costs for the schizophrenia population were estimated to be varied between US$94 million (Puerto Rico) and US$102 billion (US), and indirect costs accounted for more than 50% of the total costs (3). In China, according to a recent national epidemiological investigation for mental disorders, the lifetime prevalence of schizophrenia was estimated at 0.6% (about 8.4 million people) (4). The YLDs caused by schizophrenia accounted for 2.35% of total YLDs in China in 2019 (5). A questionnaire-based investigation showed that the annual costs per case of schizophrenia in China amounted to US$2,586.21, which could be seen as a significant economic burden for chronic schizophrenic patients and their families (6).

Antipsychotics are the mainstay of pharmacological treatment for schizophrenia patients to alleviate psychotic symptoms and improve prognosis. First-generation antipsychotics (FGAs), such as chlorpromazine and haloperidol, have been shown to be effective; but their adverse effects, such as extrapyramidal symptoms (EPS) and tardive dyskinesia in some cases, often limit long-term adherence (7). Second-generation antipsychotics (SGAs), including clozapine, risperidone, olanzapine and aripiprazole, have been recommended as first-line treatment by national guidelines (8, 9) as having equal or better efficacy, and lower risk of EPS and tardive dyskinesia comparing to FGAs. However, SGAs have also been demonstrated to be associated with an increased risk of weight gain and other metabolic abnormalities (10–12), which frequently lead to discontinuation and/or cycling between different therapies (13–16).

Lurasidone, a new SGA, was approved by China National Medical Products Administration (NMPA) for the treatment of schizophrenia in January 2019. To date, several multicenter, double-blind, phase III studies have demonstrated that lurasidone was associated with significant improvements in symptom reduction and minimal changes in weight, body mass index, and metabolic outcomes vs. placebo and quetiapine (17–20). Moreover, indirect comparison studies evaluating the efficacy and safety profile of atypical antipsychotics indicated that lurasidone was associated with significant improvements in terms of weight gain, metabolic outcomes, relapse rates, hospitalizations, and rates of all-cause discontinuation compared with olanzapine, risperidone, and aripiprazole (21, 22).

Although the clinical effectiveness of lurasidone in the treatment of schizophrenia has been demonstrated, the cost-effectiveness of lurasidone vs. alternative therapies remains to be established. This study aims to assess the cost-effectiveness of lurasidone compared with olanzapine and risperidone, which are the most prescribed SGAs in China and have been incorporated into the National Reimbursement Drug List (NRDL), in patients with schizophrenia from a Chinese healthcare system perspective. Considering that this study was the first to evaluate the cost-effectiveness of lurasidone for the treatment of schizophrenia after the drug pricing negotiation conducted by the National Healthcare Security Administration (NHSA) in 2020 (the latest negotiated price was used), the results may help inform updated clinical decisions related to schizophrenia in China.

## Materials and methods

### Model overview

**Figure 1:**
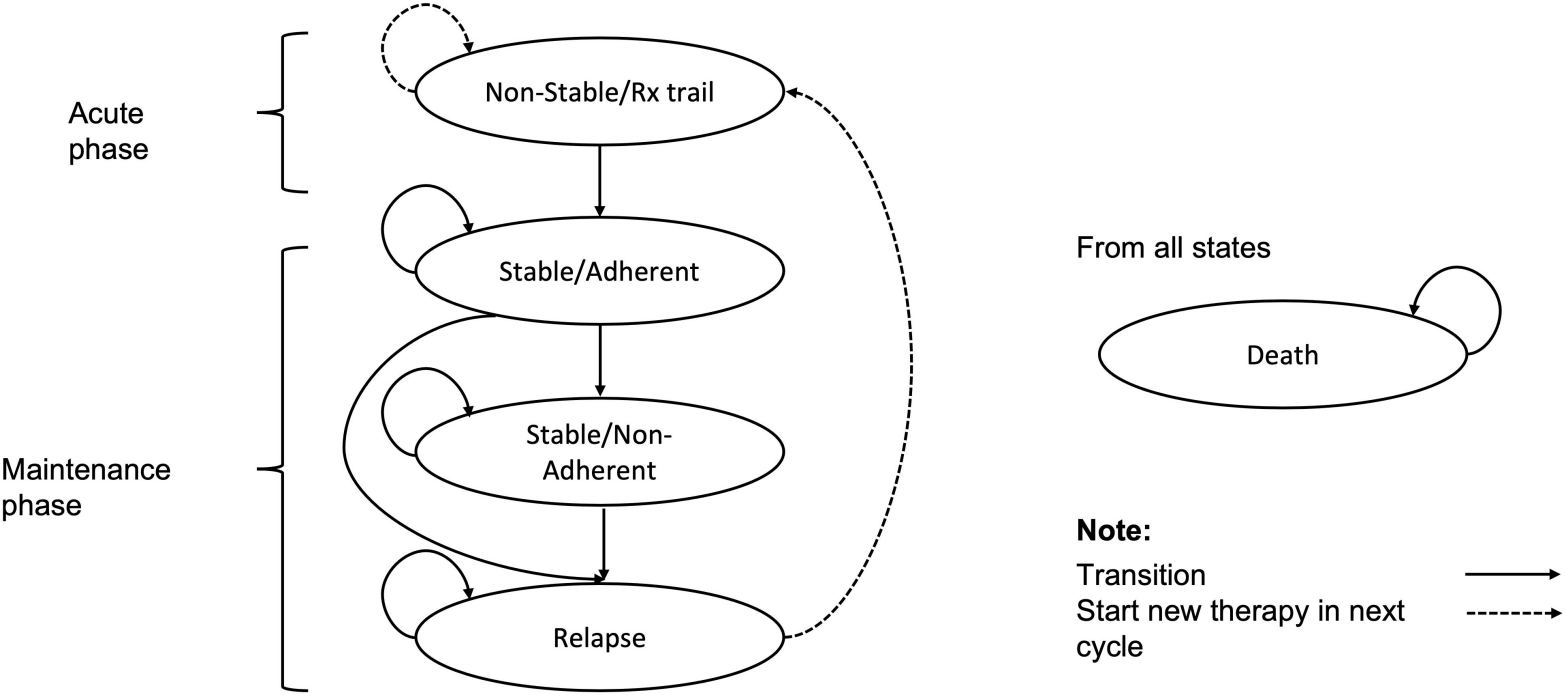
Markov model structure.

In this study, a Markov model was constructed to simulate costs and health outcomes in a hypothetical cohort of patients with schizophrenia, which had been previously developed to compare the cost-effectiveness of lurasidone with aripiprazole for the treatment of schizophrenia in the Scotland and Wales setting (23) (Figure 1). Given the chronic nature of schizophrenia, a 15-year horizon was used in the model as it was considered sufficient and recommended by Chinese clinicians to assess the long-term impact of treatment. A model cycle length of 6-week was used to reflect the clinically meaningful amount of time for the progression of schizophrenia and align with the short-term clinical trial design of lurasidone (17).

The model consisted of five health states: (1) non-stable/Rx trial, (2) stable/adherent, (3) stable/non-adherent, (4) relapse, and (5) death. Patients entered the model in the ‘non-stable/Rx trial' health state (an acute phase of relapse undergoing trials of antipsychotic agents). After 6 weeks, patients who have not discontinued treatment were assumed to enter the ‘stable/adherent' health state (the maintenance phase), while those who have discontinued treatment for any reason were assumed to switch therapy and re-enter the ‘non-stable/Rx trial' health state to continue the process of trialing alternative antipsychotic agents. Patients in the ‘stable/adherent' health state in the maintenance phase were further subject to risks of all-cause discontinuation and relapse. Patients discontinuing treatment in the maintenance phase were assumed to receive no therapy, and reside in the ‘stable/non-adherent' health state until the onset of relapse, at which point they enter the ‘relapse' health state. Patients who relapse were assumed to discontinue current therapy and switch to the next therapy, and most relapse patients were hospitalized. Patients may also die from any health state within the model.

This analysis was conducted from a Chinese healthcare system perspective. Costs and health outcomes were discounted at a rate of 5% per year in accordance with the recommendation of the China Guidelines for Pharmacoeconomic Evaluations (2020) (24).

### Patients and treatment sequences

The population for the model included adult patients diagnosed with schizophrenia. Patient characteristics were specified to reflect the average schizophrenia patient enrolled in the 6-week, randomized, double-blind, placebo- and active-controlled lurasidone clinical trial (17): 68.3% of patients were male, with age of 37.2 years old and weight of 74.5 kg.

The model compared three alternative treatment sequences. Based on the Chinese guidelines for the prevention and treatment of schizophrenia and the opinions of Chinese clinical experts, simplified treatment sequences were constructed. The first strategy consisted of lurasidone, followed by aripiprazole, clozapine and, finally, an augmented clozapine strategy (clozapine combined with risperidone). The subsequent treatment sequences of olanzapine and risperidone strategies were the same as lurasidone.

### Clinical inputs

A 2019 published systematic review and network meta-analysis (NMA) of 32 oral antipsychotics (22), including lurasidone, olanzapine, and risperidone vs. placebo, was used to inform estimates of short-term efficacy (probability of all-cause discontinuation) in the acute phase. The probability of all-cause discontinuation of placebo arm was derived from the 6-week lurasidone clinical trial (17). The published NMA did not report long-term clinical outcomes, and no other comparative clinical data were available for lurasidone vs. olanzapine and risperidone. Therefore, for the maintenance phase of the model, long-term risks of relapse and all-cause discontinuation for lurasidone were taken from a 12-month, randomized, double-blind, active-controlled study vs. quetiapine (20). To inform the olanzapine and risperidone data, the quetiapine arm of the lurasidone trial was used as the common comparator of the indirect comparison, with hazard ratios (HR) for risks of treatment discontinuation taken from a published observational study (25) and for risks of relapse taken from a mixed treatment comparison conducted by National Institute for Health and Care Excellence (NICE) (26).

Additionally, in the acute phase, patients cycled through a variety of treatment regimens until they reached a stable disease state. The efficacy data of subsequent therapies (aripiprazole, clozapine, and augmented clozapine) were taken from the published NMA (22). Data for augmented clozapine were assumed to equal the data for clozapine. For the maintenance phase, the HR of aripiprazole vs. quetiapine on the risk of discontinuation was taken from the published observational study (25), and the HR of aripiprazole vs. quetiapine on the risk of relapse was taken from the NICE mixed treatment comparison (26). In the absence of data, the risk of relapse and discontinuation of clozapine and augmented clozapine were assumed to be equal to quetiapine in the maintenance phase. The proportion of relapse attributed to adherent patients was derived from a Chinese real-world study (27).

With respect to the safety data, weight gain (defined as a ≥7% change in weight from baseline), EPS and diabetes were taken into account in this study. Incidences of weight gain and EPS for diverse antipsychotics were derived from the short-term lurasidone clinical trial (the placebo arm was used) (17) and the published NMA (22). The incidence of diabetes was estimated based on a cost-effectiveness analysis of eleven antipsychotics in Singapore (the olanzapine arm was used) (28) and an economic evaluation conducted by NICE (the relative effect of developing diabetes was assumed to be equal to the relative effect of experiencing weight gain) (26).

Mortality was based on the Chinese life table of the general population (29) and adjusted by the standard mortality rates of Chinese schizophrenia patients (30). A summary of model clinical inputs is provided in Table 1.

**Table 1:** Summary of clinical data used in the model.

**Variable**	**Base-case value**	**OWSA**		**PSA**	**Source**
		**Lower value**	**Upper value**	**distribution**	
All-cause discontinuation in non-stable/Rx trial state					
Placebo	39.34%	NA^a^	NA^a^	NA^a^	(17)
Lurasidone (RR vs. placebo)	0.88	0.80	0.96	Log-normal	(22)
Olanzapine (RR vs. placebo)	0.69	0.65	0.74	Log-normal	(22)
Risperidone (RR vs. placebo)	0.83	0.80	0.85	Log-normal	(22)
Aripiprazole (RR vs. placebo)	0.80	0.73	0.86	Log-normal	(22)
Clozapine (RR vs. placebo)	0.75	0.59	0.91	Log-normal	(22)
Augmented clozapine (RR vs. placebo)	0.75	0.59	0.91	Log-normal	Assumption
All-cause discontinuation in stable/adherent state					
Quetiapine	Weibull	NA^a^	NA^a^	NA^a^	(20)
Lurasidone (HR vs. quetiapine)	0.72	0.52	1.02	Log-normal	(20)
Olanzapine (HR vs. quetiapine)	0.74	0.55	0.92	Log-normal	(25)
Risperidone (HR vs. quetiapine)	1.16	0.87	1.45	Log-normal	(25)
Aripiprazole (HR vs. quetiapine)	0.87	0.65	1.09	Log-normal	(25)
Clozapine (HR vs. quetiapine)	1.00	0.75	1.25	Log-normal	Assumption
Augmented clozapine (HR vs. quetiapine)	1.00	0.75	1.25	Log-normal	Assumption
Relapse in stable state					
Quetiapine	Gompertz	NA^a^	NA^a^	NA^a^	(20)
Lurasidone (HR vs. quetiapine)	0.70	0.39	1.24	Log-normal	(20)
Olanzapine (HR vs. quetiapine)	0.69	0.52	0.87	Log-normal	(26)
Risperidone (HR vs. quetiapine)	1.00	0.75	1.25	Log-normal	(26)
Aripiprazole (HR vs. quetiapine)	0.99	0.75	1.24	Log-normal	(26)
Clozapine (HR vs. quetiapine)	1.00	0.75	1.25	Log-normal	Assumption
Augmented clozapine (HR vs. quetiapine)	1.00	0.75	1.25	Log-normal	Assumption
Proportion of relapse from adherent patients	38.20%	28.65%	47.75%	Beta	(27)
AE of weight gain					
Placebo	3.29%	NA^a^	NA^a^	NA^a^	(17)
Lurasidone (RR vs. placebo)	1.29	0.97	1.61	Log-normal	(22)
Olanzapine (RR vs. placebo)	6.10	4.58	7.63	Log-normal	(22)
Risperidone (RR vs. placebo)	2.83	2.12	3.54	Log-normal	(22)
Aripiprazole (RR vs. placebo)	1.50	1.13	1.88	Log-normal	(22)
Clozapine (RR vs. placebo)	10.91	8.18	13.64	Log-normal	(22)
Augmented clozapine (RR vs. placebo)	10.91	8.18	13.64	Log-normal	Assumption
AE of EPS					
Placebo	3.00%	NA^a^	NA^a^	NA^a^	(17)
Lurasidone (RR vs. placebo)	1.92	1.43	2.50	Log-normal	(22)
Olanzapine (RR vs. placebo)	1.02	0.79	1.28	Log-normal	(22)
Risperidone (RR vs. placebo)	1.79	1.41	2.38	Log-normal	(22)
Aripiprazole (RR vs. placebo)	1.33	0.90	1.82	Log-normal	(22)
Clozapine (RR vs. placebo)	0.46	0.19	0.88	Log-normal	(22)
Augmented clozapine (RR vs. placebo)	0.46	0.19	0.88	Log-normal	Assumption
AE of diabetes					
Olanzapine	0.69%	NA^a^	NA^a^	NA^a^	(28)
Lurasidone (RR vs. olanzapine)	0.21	0.16	0.26	Log-normal	(22)
Risperidone (RR vs. olanzapine)	0.46	0.35	0.58	Log-normal	(22)
Aripiprazole (RR vs. olanzapine)	0.25	0.19	0.31	Log-normal	(22)
Clozapine (RR vs. olanzapine)	1.79	1.34	2.24	Log-normal	(22)
Augmented clozapine (RR vs. olanzapine)	1.79	1.34	2.24	Log-normal	Assumption
SMR male	10.17	7.63	12.71	Log-normal	(30)
SMR female	12.42	9.32	15.53	Log-normal	(30)

a Variable not included in the sensitivity analysis.

AE, adverse event; EPS, extrapyramidal symptoms; HR, hazard ratio; OWSA, one-way sensitivity analysis; PSA, probabilistic sensitivity analysis; RR, risk ratio; SMR, standardized mortality ratio.

### Costs and resource utilization inputs

From the perspective of Chinese healthcare system, resource use included drug acquisition, schizophrenia related outpatient visits, schizophrenia related inpatient visits, and adverse events (AEs) treatment.

Due to the lack of data, a face-to-face survey of clinical experts was conducted to understand the healthcare resource utilization related to standard schizophrenia treatment and AEs treatment. To be eligible, clinical experts had to be working in tertiary hospital (where the majority of schizophrenia patients are treated), have more than 5 years of practical experience and be providing treatments for individual patients. A total of 5 clinical experts were selected, one each from Shenyang, Beijing, Chengdu, Shanghai, and Changsha. This was done to consider the different geographic areas and economic development in China.

The unit costs of antipsychotics were the most recent average bidding prices in all available provinces in China, which could be queried through the Chinese open-source Yaozh website (31). The daily dosages were consistent with the instructions of each drug (Table 2). According to the expert survey, patients with either non-stable disease, stable disease or relapse disease were required to take regular outpatient visits including tests for liver function, kidney function, blood routine, blood biochemistry, electrocardiogram, etc. The unit cost of those healthcare resources was acquired from the governmental publications in five cities where the clinical experts come from (32–36). Patients who experienced non-stable disease, stable disease and relapse disease were 52.0, 0.6, and 41.0% possible to be hospitalized, and the average hospital stay of those patients was 26.4, 3.8, and 31.0 days, respectively. Inpatient costs were then calculated via average inpatient days multiplied by inpatient daily cost, which could be found in Table 2. The treatment costs of AEs, including weight gain and EPS, were estimated by the expert survey. Specifically, the use of healthcare resources was described by clinicians, and the unit price of those healthcare resources was obtained from the governmental publications (32–36). The average annual cost for diabetes treatment was derived from a multicenter, prospective cohort study in China (37), and adjusted to 6-week cost to fit the model cycle length. All costs were expressed in 2020 Chinese Yuan (CN¥) and US$ [average exchange rate in 2020: US$1 = CN¥6.90 (38)]. A summary of the cost data in the model is presented in Table 2.

**Table 2:** Summary of cost data used in the model.

**Variable**	**Base-case value**	**OWSA**		**PSA**	**Source**
		**Lower value**	**Upper value**	**distribution**	
Drug acquisition costs					
Daily dosage, mg					
Lurasidone	60.00	NA^a^	NA^a^	NA^a^	Drug instruction
Olanzapine	12.50	NA^a^	NA^a^	NA^a^	Drug instruction
Risperidone	5.00	NA^a^	NA^a^	NA^a^	Drug instruction
Aripiprazole	20.00	NA^a^	NA^a^	NA^a^	Drug instruction
Clozapine	150.00	NA^a^	NA^a^	NA^a^	Drug instruction
Unit cost per dosage, CN¥ (US$)/mg					
Lurasidone	0.240 (0.035)	0.180	0.300	Gamma	(31)
Olanzapine	1.549 (0.224)	1.162	1.936	Gamma	(31)
Risperidone	0.635 (0.092)	0.476	0.794	Gamma	(31)
Aripiprazole	0.787 (0.114)	0.590	0.984	Gamma	(31)
Clozapine	0.001 (0.0001)	0.001	0.001	Gamma	(31)
Schizophrenia related outpatient costs, CN¥ (US$)/6-week					
Non-stable state and relapse state	615.88 (89.26)	461.91	769.85	Gamma	(32–36)
Stable state	312.48 (45.29)	234.36	390.60	Gamma	(32–36)
Schizophrenia related inpatient costs					
Duration, days					
Non-stable state and relapse state	26.40	19.80	33.00	Log-normal	Expert survey
Stable state	3.80	2.85	4.75	Log-normal	Expert survey
Relapse state	31.00	23.25	38.75	Log-normal	Expert survey
Daily cost, CN¥ (US$)/day					
Non-stable state and relapse state	520.00 (75.36)	390	650	Gamma	(32–36)
Stable state	240.00 (34.78)	180	300	Gamma	(32–36)
Relapse state	520.00 (75.36)	390	650	Gamma	(32–36)
AEs management costs, CN¥ (US$)/6-week					
Weight gain	78.62 (11.39)	58.97	98.28	Gamma	(32–36)
EPS	100.98 (14.63)	75.74	126.23	Gamma	(32–36)
Diabetes	1,544.83 (223.89)	1,158.62	1,931.04	Gamma	(37)

a Variable not included in the sensitivity analysis.

AE, adverse event; EPS, extrapyramidal symptoms; OWSA, one-way sensitivity analysis; PSA, probabilistic sensitivity analysis.

### Utility inputs

Utility values of schizophrenia states and utility decrements associated with AEs were mainly obtained from a direct utility elicitation study (39). The specific utility and disutility values adopted in the model are shown in Table 3.

**Table 3:** Summary of utility data used in the model.

**Variable**	**Base-case value**	**OWSA**		**PSA**	**Source**
		**Lower value**	**Upper value**	**distribution**	
Health state utility values					
Stable	0.919	0.874	0.964	Beta	(39)
Non-stable/relapse	0.604	0.522	0.686	Beta	(39)
AE-related disutility values					
Weight gain	0.089	0.052	0.126	Beta	(39)
EPS	0.256	0.227	0.285	Beta	(39)
Diabetes	0.151	0.135	0.167	Beta	(39)

AE, adverse event; EPS, extrapyramidal symptoms; OWSA, one-way sensitivity analysis; PSA, probabilistic sensitivity analysis.

### Base-case and sensitivity analyses

In the base-case analysis, total costs, and total numbers of quality-adjusted life years (QALYs) associated with lurasidone, olanzapine and risperidone over 15 years were estimated. Incremental cost-effectiveness ratios (ICERs) were also calculated, presented as incremental cost per QALY gained. Conventionally, the willingness-to-pay threshold was 1–3 times of Chinese per capita gross domestic product (GDP), namely CN¥72,447.00 (US$10,499.57)–CN¥2,17,341.00 (US$31,498.70) in 2020 (38).

Robustness of the results of this analysis was tested by one-way sensitivity analyses (OWSA) and probabilistic sensitivity analyses (PSA). In OWSA, the discount rates for costs and health outcomes were varied between 0 and 8% per annum (24), while other key parameters were varied by 95% confidence intervals or ±25% of the base-case values (when confidence intervals were not available). The net monetary benefit (NMB), assuming the willingness-to-pay threshold of CN¥72,447.00 (US$10,499.57) per QALY (one time of Chinese per capita GDP), was calculated at the upper and lower parameter values and was used to plot a tornado diagram. Monte Carlo simulation was used to conduct the PSA. All key parameters were assigned distributions and varied simultaneously over 5,000 iterations. The results of PSA were plotted on a cost-effectiveness acceptability curve. The specific values of parameters used in OWSA, and parameter distributions used in PSA are presented in Tables 1–3.

## Results

### Base-case analysis

Table 4 presents the results of the base-case analysis. Compared with olanzapine and risperidone, lurasidone was the dominant strategy associated with reduced costs and increased QALYs. Over a 15-year time horizon, the total cost of patients treated with lurasidone was CN¥128,662 (US$18,647), CN¥12,093 (US$1,753) lower than that of patients treated with olanzapine, and CN¥6,781 (US$983) lower than that of patients treated with risperidone. Total QALYs of patients treated with lurasidone were 8.147, 0.197 higher than those of patients treated with olanzapine, and 0.116 higher than those of patients treated with risperidone.

**Table 4:** Results of the base-case analysis.

**Treatment**	**Total costs, CN¥(US$)**	**Total QALYs**	**Incremental costs, CN¥(US$)**	**Incremental QALYs**	**ICER, CN¥(US$)/ QALY**
Lurasidone	128,662 (18,647)	8.147	—	—	—
Olanzapine	140,755 (20,399)	7.950	−12,093 (-1,753)	0.197	Lurasidone dominant
Risperidone	135,443 (19,629)	8.031	−6,781 (-983)	0.116	Lurasidone dominant

QALY, quality-adjusted life-years; ICER, incremental cost-effectiveness ratio.

### Sensitivity analyses

**Figure 2:**
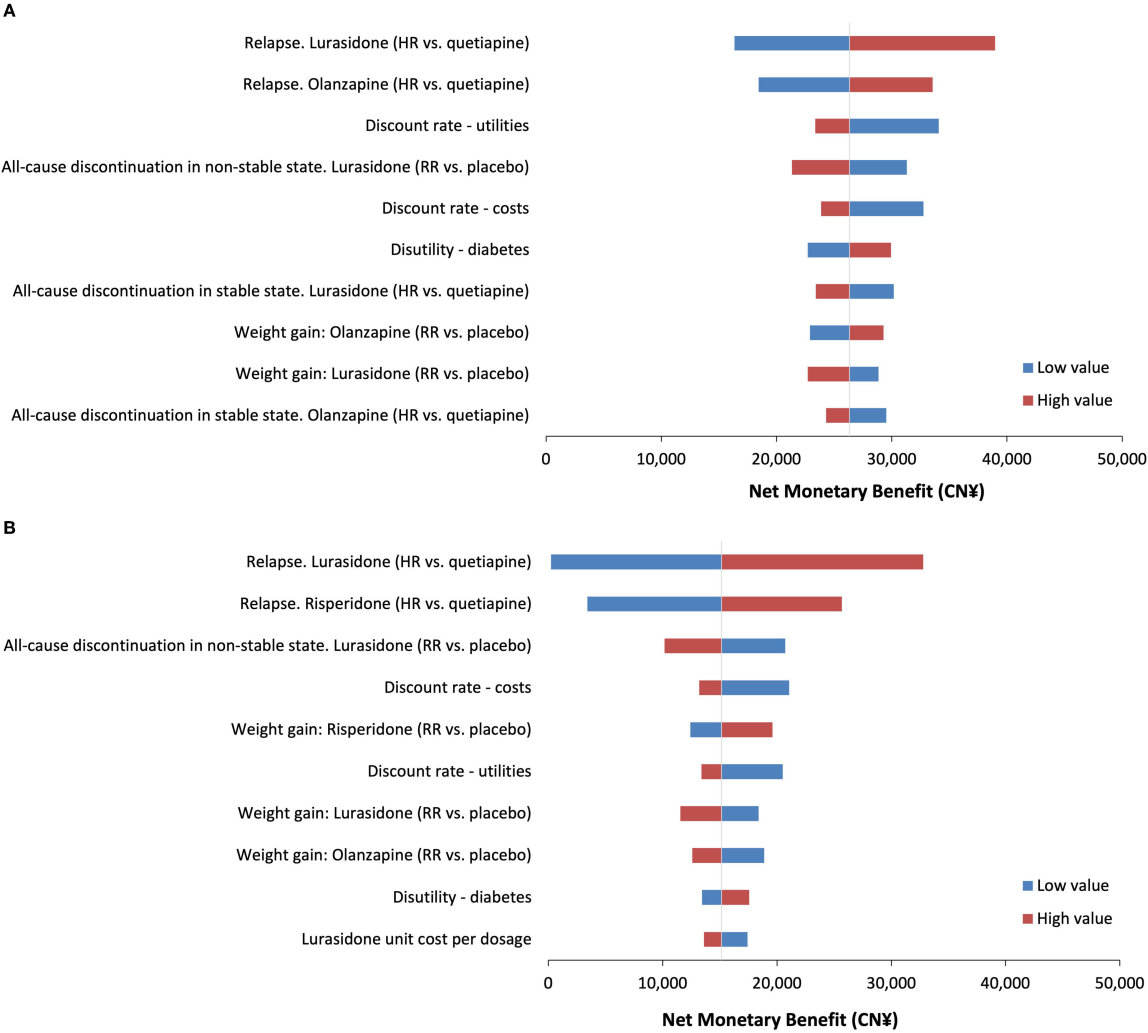
Tornado diagram for one-way sensitivity analyses. **(A)** The net monetary benefit for lurasidone vs. olanzapine. **(B)** The net monetary benefit for lurasidone vs. risperidone. HR, hazard ratio; RR, risk ratio.

The OWSA revealed that the model parameter with the most impact on the cost-effectiveness of lurasidone vs. olanzapine was the relapse HR for lurasidone vs. quetiapine, with the NMB ranging from CN¥16,355 (US$2,370) to CN¥38,968 (US$5,648). Other influential parameters were the relapse HR for olanzapine vs. quetiapine and the discount rate of utilities. For all OWSA results, NMBs remained >0. Similar results were observed when assessing the cost-effectiveness of lurasidone compared with risperidone. The NMB ranged from CN¥2,38 (US$34) to CN¥32,790 (US$4,752) when the relapse HR for lurasidone vs. quetiapine varied by the 95% confidence interval. The results of OWSA comparing lurasidone with olanzapine and lurasidone with risperidone are shown in Figure 2, with the top 10 influential parameters presented in the tornado diagram.

**Figure 3:**
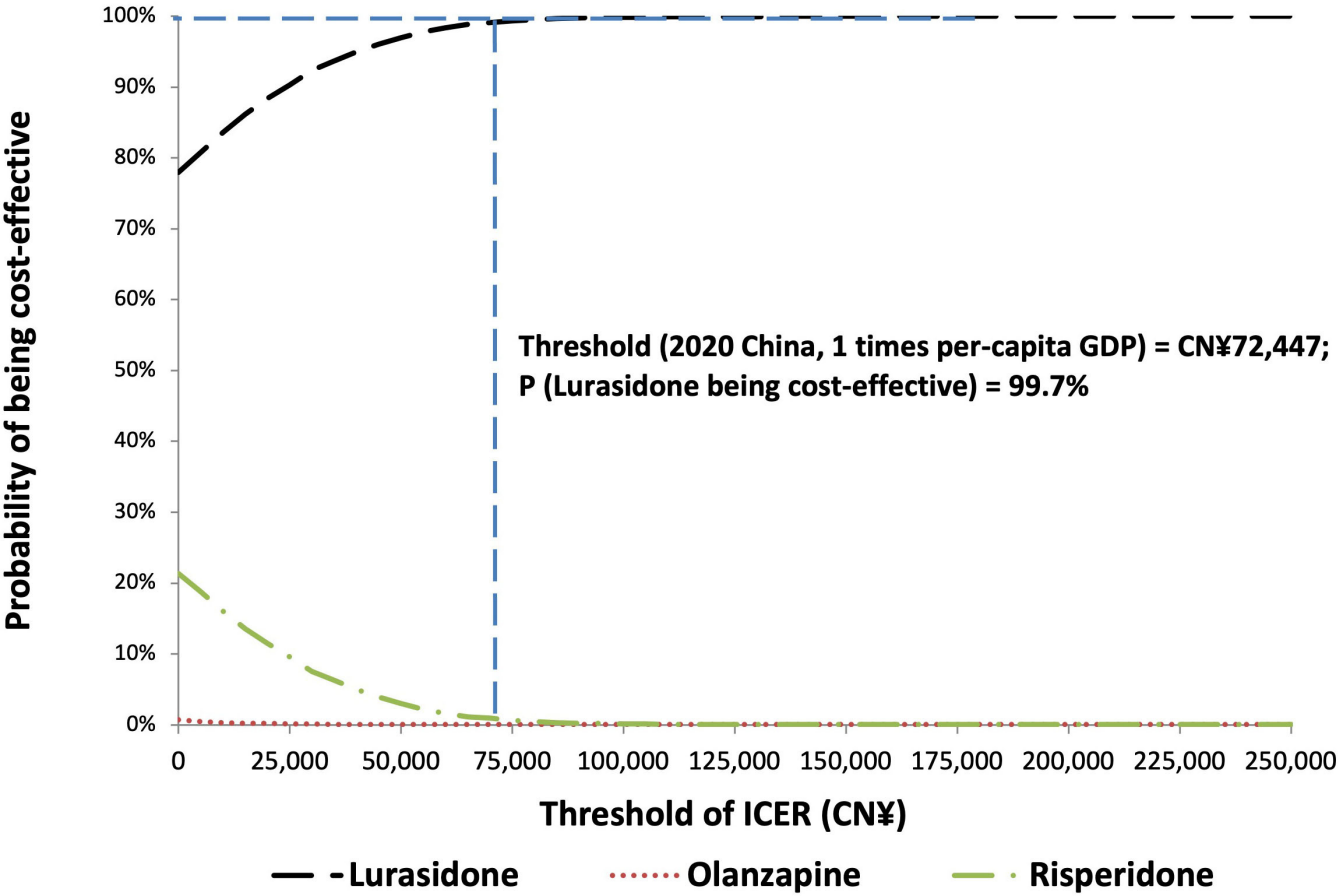
Cost-effectiveness acceptability curve for probabilistic sensitivity analysis. GDP, gross domestic product; ICER, incremental cost-effectiveness ratio.

The PSA of 5,000 simulations also showed lurasidone to be cost-effective compared with either olanzapine or risperidone at all willingness-to-pay thresholds. The probabilities that lurasidone was the cost-effective strategy were 99.7, 99.9, and 100% at the willingness-to-pay thresholds of 1, 2, and 3 times of Chinese per capita GDP in 2020 [namely CN¥72,447.00 (US$10,499.57)/QALY, CN¥1,44,894.00 (US$20,999.13)/QALY, and CN¥2,17,341.00 (US$31,498.70)/QALY], respectively. The results of PSA are presented in the cost-effectiveness acceptability curve (Figure 3).

## Discussion

In recent years, the NHSA of China has been incorporating drugs into NRDL through the drug pricing negotiation, to improve the availability and affordability of patented drugs for patients and optimize the structure of NRDL. Lurasidone was incorporated into China NRDL through the drug pricing negotiation in 2020, with the drug price decrease of 82.7%. To the best of our knowledge, this study, using the latest NRDL-negotiated price of lurasidone, is the first economic evaluation of lurasidone in treating patients with schizophrenia in China.

In this study, a published Markov model was applied to assess the cost-effectiveness of lurasidone vs. olanzapine and risperidone in China from a healthcare system perspective. Findings of this analysis suggested that, compared with these two commonly prescribed antipsychotics, lurasidone was found to be a dominant strategy associated with greater QALY gains at lower costs. The results were mainly attributed to the lower risk of weight gain of lurasidone than olanzapine and risperidone, which led to a lower risk of developing diabetes and a lower cost of AEs treatment. A variety of OWSA and PSA demonstrated the robustness of base-case results, and all sensitivity analyses yielded NMBs >0 at the strictest willingness-to-pay threshold of CN¥72,447.00 (US$10,499.57)/QALY.

Economic evaluations evaluating lurasidone vs. other available atypical antipsychotics have been conducted in a few countries. One study from a US payer perspective evaluated the cost-effectiveness of lurasidone compared with risperidone, olanzapine, ziprasidone, aripiprazole, and quetiapine through a 5-year Markov model (40). Health states included in the model were patients: on an initial atypical antipsychotic; switched to a second atypical antipsychotic; and on clozapine after failing a second atypical antipsychotic. The results showed olanzapine, ziprasidone, aripiprazole, and quetiapine were dominated by other comparators and removed from the comparative analysis, and lurasidone was cost-effective at willingness-to-pay thresholds of >US$25,844 per hospitalization avoided compared with risperidone. Another study from the perspective of Scotland and Wales healthcare services evaluated the cost-effectiveness of lurasidone vs. aripiprazole through a 10-year Markov model (23), the structure of which was adopted in the present study. The findings of the prior study suggested that lurasidone was a dominant strategy, with an increase of 0.005 QALYs and cost savings of £3,383 in Scotland and £3,072 in Wales. Thus, previous studies and our study are consistent in demonstrating the economic advantages of lurasidone compared with other atypical antipsychotics in treating patients with schizophrenia over a variety of time horizon.

There are some limitations to this study that should be considered when interpreting its results. First, to compare lurasidone vs. olanzapine and risperidone in the model, indirect comparisons were used to inform the clinical efficacy and safety. While healthcare decision-makers increasingly recognize indirect comparisons as an acceptable alternative method of comparison in the absence of real-world parallel-group data, differences in study populations may limit their comparability. Therefore, future studies evaluating the cost-effectiveness of lurasidone compared with olanzapine and risperidone based on direct comparison data are needed to verify the findings of this study. Second, due to the lack of data, a face-to-face survey of clinical experts was conducted to understand the healthcare resource utilization related to standard schizophrenia treatment and AEs treatment, which may lead to the uncertainty associated with schizophrenia-related outpatients, inpatient, and AEs treatment costs. Nonetheless, sensitivity analyses showed that changes in these costs had limited effect on the ICER value. Third, utility values used in this study were obtained from foreign studies as we did not identify available data on Chinese schizophrenia patient. As discussed in a recent publication, applying utility values derived from the previous studies to cost-utility analyses may result in the heterogeneity among results, which might be impacted by the differences in survey responders, elicitation methods, and regions (41). We therefore tested model utility parameters in sensitivity analyses and found that these values did not have a major impact on the study results. However, caution should be taken when extrapolating our findings to other health systems, as all model inputs in this study were specific to the Chinese healthcare setting. Finally, one limitation of our analysis is that it relies on the *post-hoc* analysis of clinical trials, in which assessing economic value is rarely the primary purpose. Since the results of this study could be regarded as preliminary, it will be important to further explore the cost-effectiveness of lurasidone in China based on the real-world evidence or to conduct an economic evaluation alongside the clinical trial of lurasidone.

As far as this study was concerned, compared with olanzapine and risperidone, lurasidone was a dominant strategy that yield more QALY gains with lower costs for the first-line treatment of schizophrenia in China. The robustness of the results was verified by sensitivity analyses. As the first analysis accessing the cost-effectiveness of lurasidone in China, the results may assist to fill gaps in clinical decisions regarding pharmacotherapies of schizophrenia.

## Data availability statement

The original contributions presented in the study are included in the article/supplementary material, further inquiries can be directed to the corresponding author/s.

## Author contributions

JL, LC, and JW contributed to conception and design of the study, collection of data, and development of decision analytical model. JL and LC conducted the data analysis. All authors participated in critically reviewing and interpreting the data, reviewed the manuscript for intellectual content and approved the submitted version, and agree to be accountable for all aspects of the work.

## Funding

The authors declare that this study received funding from Sumitomo Pharma (Suzhou) Co., Ltd. The funder was not involved in the study design, collection, analysis, interpretation of data, the writing of this article, or the decision to submit it for publication.

## Conflict of interest

The authors declare that the research was conducted in the absence of any commercial or financial relationships that could be construed as a potential conflict of interest.

## Publisher's note

All claims expressed in this article are solely those of the authors and do not necessarily represent those of their affiliated organizations, or those of the publisher, the editors and the reviewers. Any product that may be evaluated in this article, or claim that may be made by its manufacturer, is not guaranteed or endorsed by the publisher.
