# Corrigendum: Cost-utility analysis of lurasidone for the first-line treatment of schizophrenia in China

**DOI:** 10.3389/fpubh.2022.1059495

**Published:** 2022-11-09

**Authors:** Jia Liu, Lidan Cao, Jing Wu

**Affiliations:** ^1^School of Pharmaceutical Science and Technology, Tianjin University, Tianjin, China; ^2^Center for Social Science Survey and Data, Tianjin University, Tianjin, China

**Keywords:** cost-utility, lurasidone, olanzapine, risperidone, schizophrenia

In the published article, there was an error. The name of the drug “risperidone” was mistakenly written as “aripiprazole” in the Abstract and Results sections.

A correction has been made to the Abstract section, Results sub-section, Paragraph 1. This sentence previously stated: “Over a 15-year time horizon, lurasidone yielded an improvement of 0.197 QALYs with a cost saving of CN¥12,093 (US$1,753) vs. olanzapine and an improvement of 0.116 QALYs with a cost saving of CN¥6,781 (US$983) vs. aripiprazole.” The corrected sentence appears below: “Over a 15-year time horizon, lurasidone yielded an improvement of 0.197 QALYs with a cost saving of CN¥12,093 (US$1,753) vs. olanzapine and an improvement of 0.116 QALYs with a cost saving of CN¥6,781 (US$983) vs. risperidone.”

Three corrections have been made to the Results section, Base-case analysis sub-section, Paragraph 1. One sentence previously stated: “Compared with olanzapine and aripiprazole, lurasidone was the dominant strategy associated with reduced costs and increased QALYs.” The corrected sentence appears below: “Compared with olanzapine and risperidone, lurasidone was the dominant strategy associated with reduced costs and increased QALYs.” Another sentence previously stated: “Over a 15-year time horizon, the total cost of patients treated with lurasidone was CN¥1,28,662 (US$18,647) and CN¥12,093 (US$1,753) lower than that of patients treated with olanzapine, and CN¥6,781 (US$983) lower than that of patients treated with aripiprazole.” The corrected sentence appears below: “Over a 15-year time horizon, the total cost of patients treated with lurasidone was CN¥128,662 (US$18,647), CN¥12,093 (US$1,753) lower than that of patients treated with olanzapine, and CN¥6,781 (US$983) lower than that of patients treated with risperidone.” The other sentence previously stated: “Total QALYs of patients treated with lurasidone were 8.147, 0.197 higher than those of patients treated with olanzapine, and 0.116 higher than those of patients treated with aripiprazole.” The corrected sentence appears below: “Total QALYs of patients treated with lurasidone were 8.147, 0.197 higher than those of patients treated with olanzapine, and 0.116 higher than those of patients treated with risperidone.”

Two corrections have been made to the Results section, Sensitivity analyses sub-section, Paragraph 1. One sentence previously stated: “Similar results were observed when assessing the cost-effectiveness of lurasidone compared with aripiprazole.” The corrected sentence appears below: “Similar results were observed when assessing the cost-effectiveness of lurasidone compared with risperidone.” The other sentence previously stated: “The results of OWSA comparing lurasidone with olanzapine and lurasidone with aripiprazole are shown in Figure 2, with the top 10 influential parameters presented in the tornado diagram.” The corrected sentence appears below: “The results of OWSA comparing lurasidone with olanzapine and lurasidone with risperidone are shown in Figure 2, with the top 10 influential parameters presented in the tornado diagram.”

A correction has been made to the Results section, Sensitivity analyses sub-section, Paragraph 2. This sentence previously stated: “The PSA of 5,000 simulations also showed lurasidone to be cost-effective compared with either olanzapine or aripiprazole at all willingness-to-pay thresholds.” The corrected sentence appears below: “The PSA of 5,000 simulations also showed lurasidone to be cost-effective compared with either olanzapine or risperidone at all willingness-to-pay thresholds.”

The authors apologize for this error and state that this does not change the scientific conclusions of the article in any way. The original article has been updated.

## Publisher's note

All claims expressed in this article are solely those of the authors and do not necessarily represent those of their affiliated organizations, or those of the publisher, the editors and the reviewers. Any product that may be evaluated in this article, or claim that may be made by its manufacturer, is not guaranteed or endorsed by the publisher.

